# Successful Management of Coronary In-Stent Restenosis: A Case Report and Literature Review

**DOI:** 10.7759/cureus.8043

**Published:** 2020-05-10

**Authors:** Syed Adeel Hassan, Noor Ul Falah, Ali Akhtar, Taha Masood, Labbeeba Shafiq

**Affiliations:** 1 Neurology, Dow University of Health Sciences, Karachi, PAK; 2 Internal Medicine, Dow University of Health Sciences, Karachi, PAK; 3 Internal Medicine, King Edward Medical University, Lahore, PAK; 4 Internal Medicine, Army Medical College, National University of Medical Sciences, Rawalpindi, PAK; 5 Accident and Emergency, King Edward Medical University, Lahore, PAK; 6 Internal Medicine, Doctors Hospital, Lahore, PAK; 7 Internal Medicine, United Medical & Dental College, Karachi, PAK

**Keywords:** in-stent restenosis, drug-eluting stent, percutaneous coronary intervention, angina, angiography, bare-metal stent

## Abstract

In-stent restenosis (ISR) arising in bare-metal stents and drug-eluting stents is difficult to manage. Herein, we report a case of ISR. Our patient had a history of percutaneous coronary intervention and presented with exaggerated angina symptoms despite being on antianginal medication. ISR was diagnosed with coronary angiography. In a clinical setting, it is treated with repeat revascularization of the blocked vessel with a re-stent placement or surgical approach.

## Introduction

In-stent restenosis (ISR) is identified on coronary vessel angiography. It is defined as a ≥50% stenosis of the coronary vessel diameter at the stent segment or its edges (5-mm segments adjacent upstream and downstream to the stent) [1,2]. The incidence of ISR with a bare-metal stent (BMS) is higher when compared with a drug-eluting stent (DES). This is because neointimal proliferation takes place more frequently in the case of BMS implantation. Restenosis has been reported in about 12% of cases of DES. It is particularly more common with the use of a first-generation DES due to late stent thrombosis. Neoatherosclerosis is another pathological phenomenon responsible for the delayed stenosis of the stent. A significant reduction has been observed in the overall occurrence of ISR with the use of DES. Therefore, a DES is safer to use and more effective than a BMS. ISR is diagnosed with coronary angiography. Currently, the treatment and management of ISR is difficult and poses a clinical challenge [1]. We report a case with ISR diagnosed with elective coronary angiography that was successfully managed with a re-stent placement.

## Case presentation

A 52-year-old female presented to our outpatient department with increasing exertional dyspnea and chest pain for the past two months. Her medical history was positive for ischemic heart disease, diabetes mellitus, and hypertension. Five years ago, she underwent cardiac catheterization followed by a percutaneous coronary intervention (PCI) with an implantation of five stents. Her ongoing medication included subcutaneous insulin, antianginals (diltiazem, nitroglycerine, ranolazine, TriCardin®), clopidogrel, and an antacid.

On presentation, the patient was afebrile with a blood pressure 162/82 mmHg, heart rate 92/min, respiratory rate 24/min, and oxygen saturation 98% on room air. Due to the worsening of her angina symptoms, she was admitted for elective coronary angiography to assess the status of previously placed stents. On admission, her labs revealed poor diabetic control (hemoglobin A1C 9.3%) and dyslipidemia (total cholesterol 214 mg/dl, triglycerides 203 mg/dl, low-density lipoprotein 165 mg/dl, very low density lipoprotein 41 mg/dl, and high-density lipoprotein 45 mg/dl). Cardiac enzymes were not raised (creatine kinase-myocardial band 19.4 U/l and troponin-I 0.44 ng/ml). Blood counts, serum electrolytes, renal and liver function tests, viral markers, and coagulation profile were within normal limits. Her previous transthoracic echocardiography was significant for hypokinesis of the inferoseptal segment of the left ventricle and an ejection fraction of 50%.

**Figure 1:**
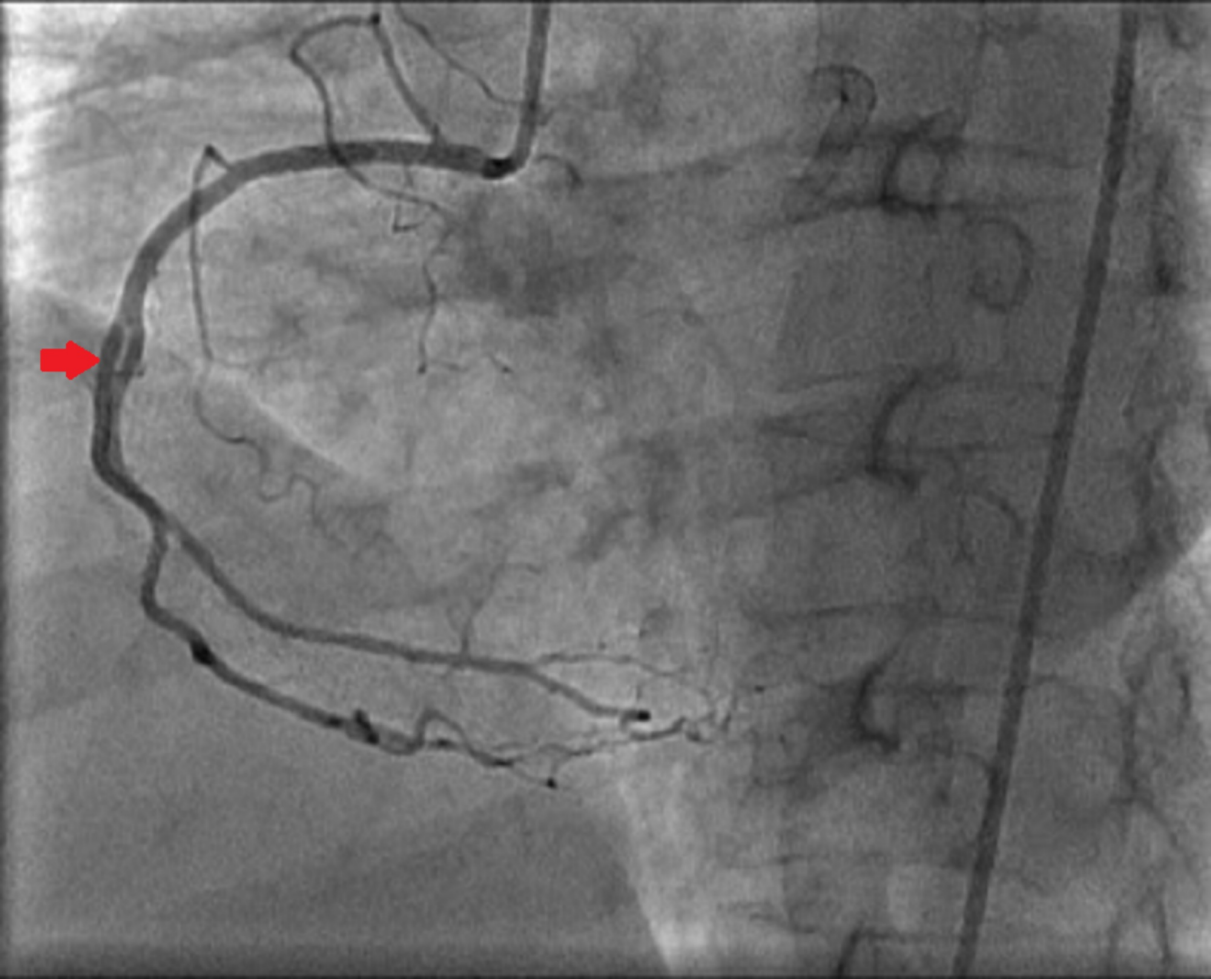
Coronary angiogram of the right coronary artery The red arrow depicts critical restenosis of the distal right coronary artery.

**Figure 2:**
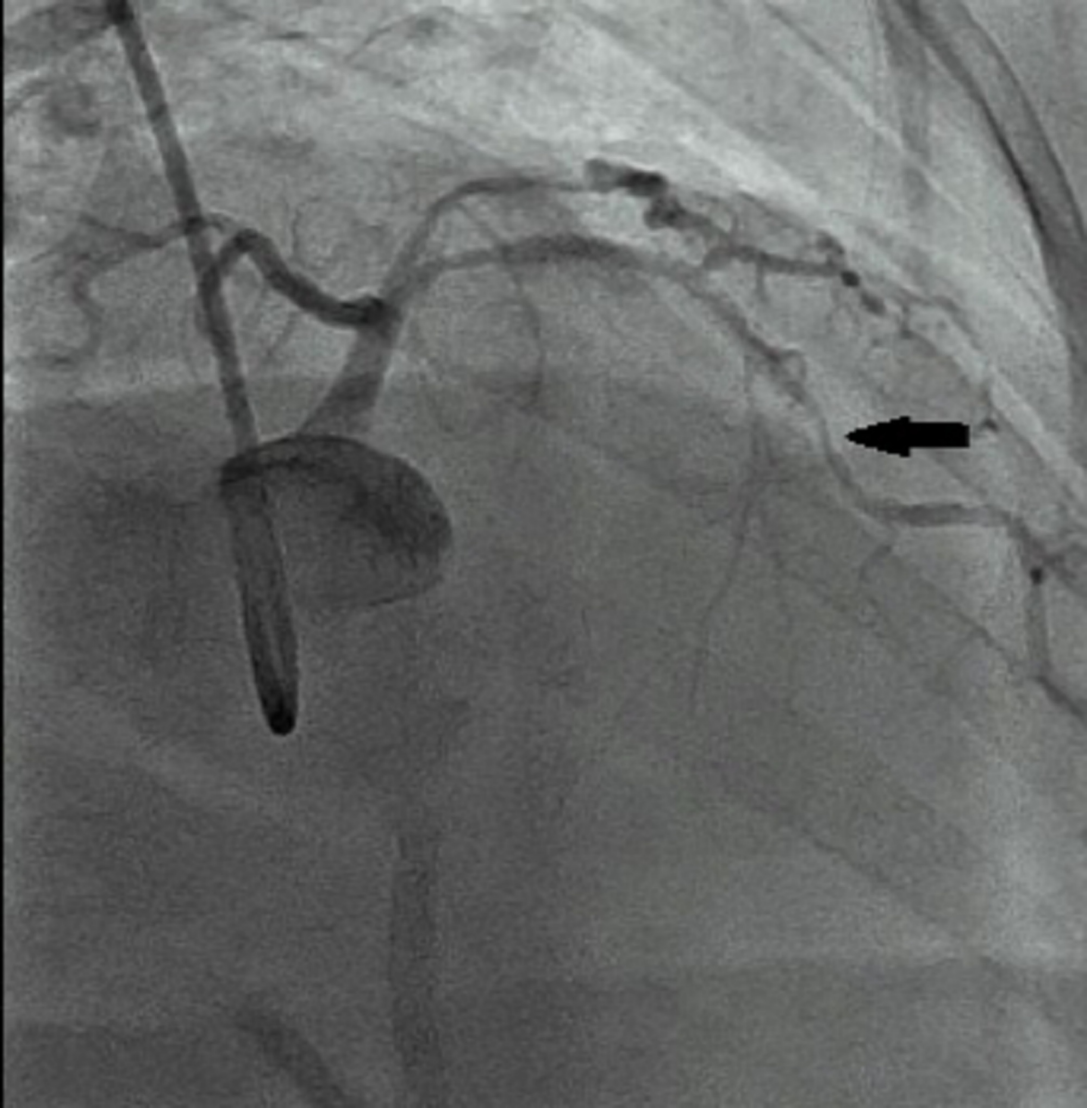
Coronary angiogram depicting the mid-LAD artery stent LAD, left anterior descending The black arrow shows in-stent restenosis of the mid-LAD artery stent.

An elective coronary angiography revealed critical ISR in the distal right coronary artery (RCA) stent (Figure 1). Furthermore, critical stenosis (80%) at the mid-distal junction of the left anterior descending (LAD) artery and 40%-50% stenosis of the proximal ostium were also seen (Figure 2). Patent stents were present in the proximal RCA and mid-LAD artery segments. The patient refused to undergo coronary artery bypass grafting. Therefore, a two-staged elective PCI involving the RCA and LAD artery was planned to treat the ISR. Stage I PCI to RCA was performed after pre-dilating the vessel. RCA ostium was intubated with guiding catheter JL3.5-6Fr. The lesion was crossed using the Hi-Torque Balance Middleweight Universal II Guidewire (Abbott Cardiovascular, Santa Clara, CA). The area was then dilated with a Mini-Trek balloon (2 x 20 mm; Abbott Cardiovascular). Finally, a Xience Xpedition® everolimus-eluting cobalt-chromium coronary stent (2.5 x 48 mm; Abbott Cardiovascular) was deployed at 17 atmospheres (ATMs) while overlapping the previous stent. The entire length of the stent was post-dilated with an NC-Trek balloon (2.75 x 15 mm; Abbott Cardiovascular). An excellent final angiographic result with well-deployed stents and thrombolysis in myocardial infarction (TIMI) III distal blood flow was achieved.

**Figure 3:**
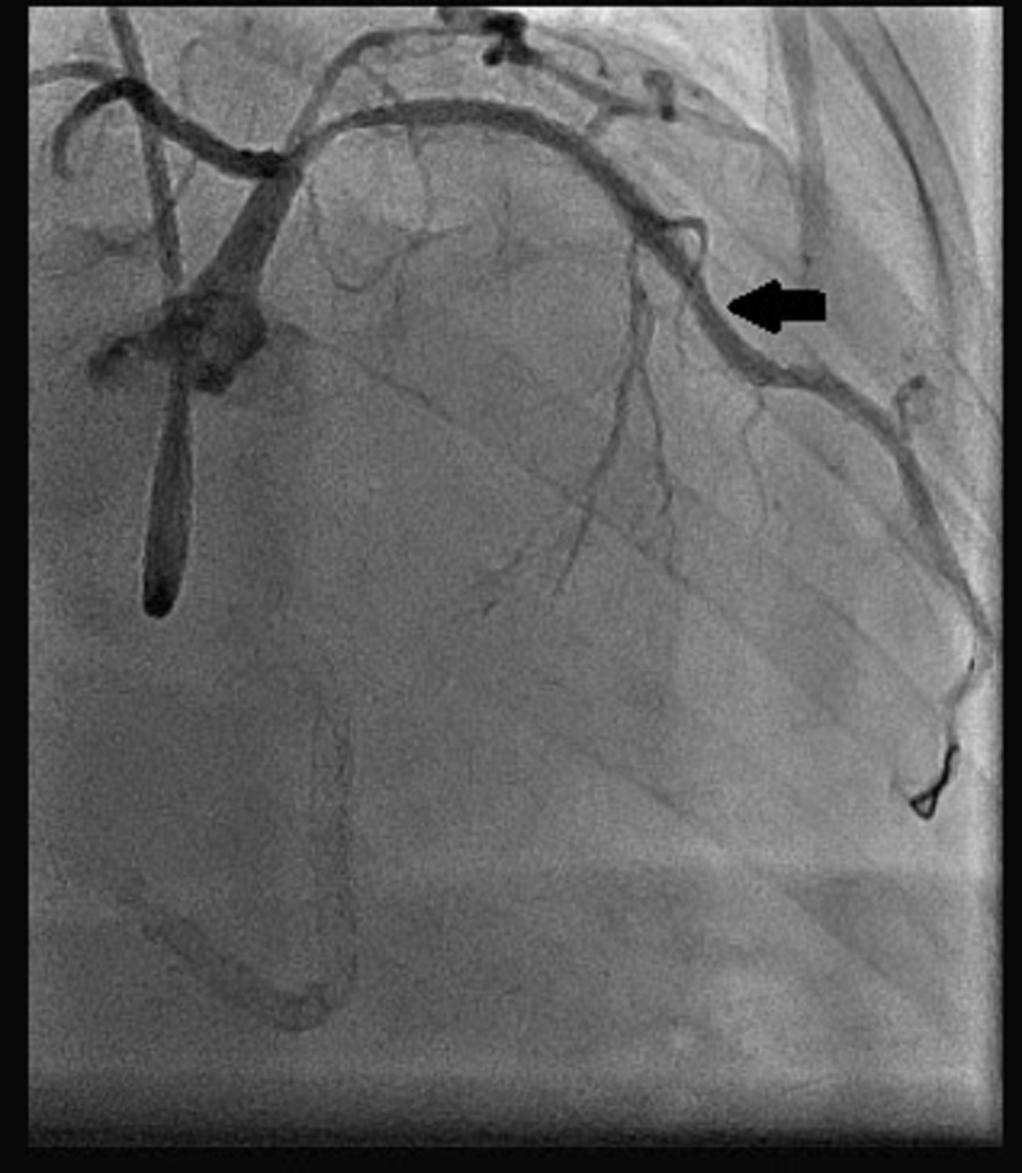
Post-stent placement coronary angiogram of the LAD artery LAD, left anterior descending; DES, drug-eluting stent The black arrow shows the reopened mid-LAD artery segment after DES placement.

Stage II PCI to the LAD artery was performed two days later. Left mainstem ostium was intubated with guiding catheter JL3.5-6Fr. The stenosed area was crossed using a Hi-Torque Balance Middleweight Universal II Guidewire. The mid-LAD artery lesion was directly stented with a Xience Prime^TM ^everolimus-eluting cobalt-chromium coronary stent (2.5 x 18 mm; Abbott Cardiovascular) and deployed at 13 ATMs overlapping the previously placed mid-LAD artery stent. Next, the overlapped segment and the previously placed stent were post-dilated with the same stent balloon at 15 ATMs. Ostial proximal LAD artery lesion was directly stented with another Xience Prime^TM^ coronary stent (2.5 x 18 mm) and deployed at 13 ATMs overlapping the first stents. Then the entire length of the stents was post-dilated with an NC-TREK balloon (2.5 x 15 mm) at 17 ATMs. An excellent final angiographic result with well-deployed stents and TIMI III distal blood flow was achieved in this case as well (Figure 3). The patient was counseled regarding risk factors and diet modification. Finally, the patient was discharged on antianginal and anti-hypertensive medication.

## Discussion

PCI is a common procedure to reopen the occluded coronary arteries [3]. Restenosis of the implanted stent segment, also known as ISR, occurs in some patients who have undergone PCI [4]. No treatment strategy has been rendered best for ISR management. Therefore, these cases are difficult to treat when encountered [2]. Patients with ongoing ISR usually present with symptoms of stable angina or asymptomatic cardiac ischemia. They clinically present 3 to 6 months after PCI [2]. Acute coronary syndrome (ACS) in the form of unstable angina occurs in about 30% to 60% of ISR cases [2,5]. Total vessel occlusion leading to ST-elevation myocardial infarction has also been reported in almost 5% of cases [2,5]. As per one study, no difference in the incidence of ACS has been found with ISR associated with either BMS or DES [5].

The underlying pathology of ISR and its angiographic morphology can be different in the case of BMS and DES [4]. However, the neointimal tissue biopsy from ISR sites in both types of stents reveals almost similar histological morphology. The occluding atheroma is mainly composed of lipids, proteoglycan-laden smooth muscle cells, and areas rich in collagen and reticular fibers [5]. The phenomena eventually leading to atheroma formation include the endothelial and smooth muscle cell proliferation, chronic inflammation of the vessel wall, macrophage activation, extracellular matrix deposition, progressive necrotic core formation, and intraplaque hemorrhages [3,4].

ISR can occur with typical balloon angioplasty of the coronary vessels and has an incidence of 30% to 60%. The major mechanisms involved are acute/chronic elastic recoil and negative constrictive remodeling of the vessel wall [2,4,5]. The recoil does not occur with the use of BMS. Neointimal hyperplasia (NIH), which is the progressive and homogeneous proliferation of smooth muscle cells, has been shown to have a strong association with lumen restenosis. NIH occurs early in the disease course followed by the regression of the proliferation and angiographically detected delayed luminal enlargement [3]. BMS-associated ISR has an incidence of about 16% to 44%. Risk factors for BMS-associated ISR include long lesion length and small vessel caliber [5]. ISR has three basic patterns based on the classification system established in 1999. This classification system is summarized in Table 1. The patterns have a prognostic value. This is because studies show that the treatment of DES-related ISR with predominant focal morphology is more effective than the treatment of BMS-related ISR with predominant nonfocal morphology [4].

**Table 1:** Classification system of ISR patterns ISR, in-stent restenosis; DES, drug-eluting stent; BMS, bare-metal stent

Focal ISR	Diffuse ISR	Occlusive ISR
This pattern is more common in DES and occurs mainly at the proximal edge of the stent, as shown in about 60% cases of ISR with either paclitaxel-eluting or sirolimus-eluting stents. Repeat treatment of BMS-associated focal ISR shows a lower recurrence risk.	This pattern is more common in BMS. The usual subtype is diffusely proliferative and involves the edges of the stent. About 20% of ISR cases in DES present a diffuse pattern. Almost one-fifth of all the ISR cases show the diffuse pattern.	DES-related ISR sometimes shows an occlusive pattern. About 10% to 20% of all the ISR cases show the occlusive pattern.

ISR is unpredictable and is associated with several risk factors and causative mechanisms. More than one factor may be involved in a single case [4]. The underlying mechanisms of ISR are shown in Table 2. It is hard to modify biological factors. However, mechanical factors can be identified and modified. Diabetes is the most frequently studied risk factor among all and is the main culprit in both BMS- and DES-associated ISR [2].

**Table 2:** Factors associated with ISR ISR, in-stent restenosis

Biological factors	Resistance to antiproliferative drugs, hypersensitivity reactions, serum matrix metalloproteinases, genetics
Angiographic/arterial factors	Wall shear stress, vessel diameter, vessel tortuosity, calcifications, thrombi, bifurcations, and chronic occlusions, venous grafts, long length (>20 mm) and ostial lesions
Patient-related factors	Diabetes mellitus, chronic renal failure/patient on dialysis, clinically unstable patients
Stent-related factors	Degree of expansion, type of eluted drug, stent fractures
Implantation factors	Barotrauma to unstented segments, geographical miss, degree of post-procedural residual stenosis

Treatment options for patients presenting with ISR include balloon angioplasty with BMS implantation, first-generation DESs, second-generation DESs, drug-coated balloons (DCBs) or surgical interventions [2]. Both second-generation DESs and DCBs are effective in treating patients with ISR. DCBs can be preferred in small-sized coronary vessels, bifurcations, and restenotic lesions with multiple stent layers. A DES can be preferred in large-sized vessels, fractured previously deployed stents, restenotic lesions with recoil, and in cases of edge dissections after balloon dilatation of the restenotic lesion. A DES has better angiographic and clinical outcomes whereas DCB eliminates the requirement for additional stent implantation [4]. The coronary artery bypass graft is reserved for patients with ISR associated with complex multivessel disease [2].

## Conclusions

ISR is associated with the use of both BMS and DES. Restenosis should be considered in a patient experiencing reappearance or worsening of angina symptoms. Coronary angiography effectively identifies ISR. Furthermore, it determines the size and extent of the restenosis. This helps better understand the underlying pathological process. Early detection and timely management of ISR helps to improve the disease outcome and prevent cardiovascular mortality in patients with atherosclerotic coronary artery disease and stent implantation.
